# Grafting Watermelon Onto Pumpkin Increases Chilling Tolerance by Up Regulating *Arginine Decarboxylase* to Increase Putrescine Biosynthesis

**DOI:** 10.3389/fpls.2021.812396

**Published:** 2022-02-15

**Authors:** Junyang Lu, Fei Cheng, Yuan Huang, Zhilong Bie

**Affiliations:** Key Laboratory of Horticultural Plant Biology, Ministry of Education, College of Horticulture and Forestry Sciences, Huazhong Agricultural University, Wuhan, China

**Keywords:** watermelon, pumpkin rootstock, grafting, transcriptome, putrescine, chilling tolerance

## Abstract

Low temperature is a major environmental factor that severely impairs plant growth and productivity. Watermelon (*Citrullus lanatus*) is a chilling-sensitive crop. Grafting of watermelon onto pumpkin rootstock is an effective technique to increase the chilling tolerance of watermelon when exposure to short-time chilling stress. However, the mechanism by which pumpkin rootstock increases chilling tolerance remains poorly understood. Under 10°C/5°C (day/night) chilling stress treatment, pumpkin-grafted watermelon seedlings showed higher chilling tolerance than self-grafted watermelon plants with significantly reduced lipid peroxidation and chilling injury (CI) index. Physiological analysis revealed that pumpkin rootstock grafting led to the notable accumulation of putrescine in watermelon seedlings under chilling conditions. Pre-treat foliar with 1 mM D-arginine (inhibitor of arginine decarboxylase, ADC) increased the electrolyte leakage (EL) of pumpkin-grafted watermelon leaves under chilling stress. This result can be ascribed to the decrease in transcript levels of *ADC*, *ornithine decarboxylase*, *spermidine synthase*, and *polyamine oxidase* genes involved in the synthesis and metabolism of polyamines. Transcriptome analysis showed that pumpkin rootstock improved chilling tolerance in watermelon seedlings by regulating differential gene expression under chilling stress. Pumpkin-grafted seedling reduced the number and expression level of differential genes in watermelon scion under chilling stress. It specifically increased the up-regulated expression of *ADC* (*Cla97C11G210580*), a key gene in the polyamine metabolism pathway, and ultimately promoted the accumulation of putrescine. In conclusion, pumpkin rootstock grafting increased the chilling tolerance of watermelon through transcription adjustments, up regulating the expression level of *ADC*, and promoting the synthesis of putrescine, which ultimately improved the chilling tolerance of pumpkin-grafted watermelon plants.

## Introduction

Low temperature is a key factor limiting not only plant growth and development but also crop quality and productivity (Thomashow, 1999; Zhu et al., 2007; Liu et al., 2018). Plants from tropical or subtropical regions are generally sensitive to chilling stress; symptoms of chilling stress include chilling injuries, such as reactive oxygen species (ROS) burst, cell membrane stiffness, ion imbalance, reduced ATP synthesis, chlorosis, growth retardation, and cell death (Ruelland and Zachowski, 2010; Seo et al., 2010; Theocharis et al., 2012). Plants have evolved sophisticated mechanisms, including physiological and biochemical modifications, to enhance chilling tolerance. Regulatory genes are involved in signal transduction and gene expression regulation, whereas functional genes serve as either direct or indirect players in chilling stress tolerance (Zhang Z. et al., 2017). Accumulation of polyamines by modulating metabolism-related genes is a useful strategy for plants to withstand low temperature-induced adverse damages, such as cell membrane permeability, osmotic potential increase, ROS accumulation and others (Alet et al., 2011; Kou et al., 2018; Chen et al., 2019).

Polyamines are a group of small aliphatic amines that are ubiquitously distributed in all living organisms (Schweikert and Burritt, 2015; Chen et al., 2019) and involved in the regulation of plant growth and development (Shi and Chan, 2014; Liu et al., 2015; Chen et al., 2019). Polyamines also improve plant tolerance to various abiotic stresses, such as drought, salt, low or high temperature, nutrient deficiency, and heavy metal stress (Liu et al., 2009; Gong et al., 2015; Sengupta and Raychaudhuri, 2017; Kou et al., 2018; Gao et al., 2020), which are valuable for the stabilization of membranes and macromolecules, detoxification of ROS, and alleviation of oxidative stress (Shi and Chan, 2014; Liu et al., 2015; Chen et al., 2019). Putrescine, spermidine and spermine are three major polyamines in plants. Plant polyamines are synthesized from arginine and ornithine with two major biosynthesis enzymes, arginine decarboxylase (ADC) and ornithine decarboxylase (ODC), degraded by copper amine oxidase and polyamine oxidase (PAO) (Shi and Chan, 2014; Liu et al., 2015). Polyamines synthesis and metabolization-related genes and enzymes are responsive to abiotic stress, generally showing a trend of polyamine accumulation and playing an important role in abiotic tolerance (Shi and Chan, 2014; Liu et al., 2015). Arginine decarboxylase is a key rate-limiting member responsible for the stress-triggered accumulation of polyamines, particularly putrescine (Kou et al., 2018; Chen et al., 2019). Putrescine accumulates in *Arabidopsis thaliana*, tomato, cucumber, potato and soybean in response to low temperature stress (Shen et al., 2000; Alet et al., 2011; Song et al., 2014a,b; Tian et al., 2015; Kou et al., 2018), and external application of putrescine significantly improves chilling tolerance in zucchini and potato (Palma et al., 2016; Kou et al., 2018). However, the role of putrescine synthesis in the chilling response of watermelon is unknown. Whether grafting watermelon onto chilling-tolerant rootstock can regulate *ADC* gene expression to modulate putrescine biosynthesis in response to chilling stress remains to be investigated.

Watermelon (*Citrullus lanatus*) is an important horticultural crop belonging to the *Cucurbitaceae* family and cultivated worldwide. Grafting is widely used in horticulture production to enhance abiotic/biotic stress resistance, promote water and fertilizer absorption, increase yield, and improve fruit quality (Lee et al., 2010; Schwarz et al., 2010; Migicovsky et al., 2019; Liu et al., 2021). Our previous research indicated that arginine biosynthesis contributes to pumpkin rootstock-induced chilling tolerance (Shi et al., 2019). However, the molecular mechanisms underlying pumpkin rootstock-mediated chilling tolerance for short-time exposure remain unclear to date. To address this question, we investigated the mechanism by which pumpkin rootstock increases chilling tolerance. We hypothesize that the pumpkin rootstock regulates scion chilling tolerance by increasing putrescine accumulation in watermelon seedlings. Our results suggest that putrescine biosynthesis is involved in the chilling tolerance regulation of grafted watermelon and provide a basis for further breeding chilling-tolerant rootstock.

## Materials and Methods

### Plant Materials and Chilling Stress Treatment

The experiment was carried out at Huazhong Agricultural University (Wuhan, China). Watermelon “97103” (*Citrullus lanatus*, a chilling-sensitive inbred line) was used as the scion, and pumpkin “Qingyan No.1” (*Cucurbita maxima* × *C. moschata*, a chilling tolerant cultivar) was used as the rootstock. The watermelon seedlings were grafted by using the “hole insertion grafting” method (Lee et al., 2010). Two grafting combinations were used in this study, including self-grafted watermelon plants (*Cl/Cl*) and pumpkin rootstock-grafted watermelon plants (*Cl/Cm*). Grafted seedlings were grown in a growth chamber at 28°C/18°C (day/night), 12 h photoperiod, photosynthetic photon flux density (PPFD) of 300 μmol⋅m^–2^⋅s^–1^, and relative humidity (RH) of 50%–70%. At the four-leaf stage, the grafted plants were used for subsequent experiments.

Three chilling treatment experiments were performed in a growth chamber (Ningbo Saifu DGX-260) with a photoperiod of 12 h of light followed by 12 h of dark and a controlled PPFD of 300 μmol⋅m^–2^⋅s^–1^. Firstly, to investigate the effects of pumpkin rootstock grafting on the chilling tolerance of watermelon seedlings, *Cl/Cl* and *Cl/Cm* seedlings were exposed to 28°C/18°C (day/night) and 10°C/5°C (day/night), respectively. At 0, 1, 3, 5, and 7 days after the chilling treatment, the leaves were collected, instantly frozen in liquid nitrogen, and then stored at −80°C for further analyses. Secondly, to investigate the effects of D-arginine (an ADC inhibitor) treatment on the chilling tolerance of pumpkin rootstock-grafted watermelon seedlings, the *Cl/Cm* seedlings were spray pretreated with 1 mM D-arginine or water (Wu et al., 2016), 12 h before exposure to chilling treatment for 3 d. The leaves were collected at the designated time points for the analysis. To further study the molecular mechanism of pumpkin rootstock grafting on chilling tolerance in watermelon seedlings, both *Cl/Cl* and *Cl/Cm* plants were exposed to 28°C/18°C (day/night) and 10°C/5°C (day/night), respectively. At 12 h after the chilling treatment, the third true leaves (picked at third node from seedling bottom) were collected for RNA sequencing and transcriptome analysis. Each treatment consisted of three replicates with six seedlings in each replicate.

### Physiological Measurements

The chilling injury index was measured as previously described (Yang et al., 2008). Briefly, the degrees of chilling damage were defined as 6 grades according to the symptom of leaf chlorosis or wilting. The chilling injury index (CI) of the *Cl/Cl* and *Cl/Cm* seedlings was calculated as follows: CI = Σ (each level × number of plants with the corresponding symptom)/total number of measured plants. The EL was measured as previously described (Cheng et al., 2016). Lipid peroxidation was determined by measuring malondialdehyde (MDA) content by thiobarbituric acid reaction method (Hodges et al., 1999). Chlorophyll fluorescence was measured using imaging PAM (MAXI, Heinz Walz, Germany), The intensities of the actinic light and saturating light settings were 280 and 4000 μmol⋅m^–2^⋅s^–1^, respectively (Cheng et al., 2016).

### Measurement of Free Polyamines Levels

Polyamines levels were measured as described previously (Gong et al., 2015). Briefly, approximately 0.1 g of fresh leaf tissue powder was extracted in 5% cold perchloric acid containing dithiothreitol (0.5 g⋅L^–1^) to extract free polyamines with 1,6-hexanediamine as an internal standard. The samples were placed on a shaker for 1 h at 4°C in the dark and then centrifuged at 12,000 rpm for 15 min at 4°C. The supernatants were taken for derivatization. Polyamines were separated with ethyl ether and centrifuged at 7100 g for 5 min. The upper phase was collected and vacuum dried in a concentrator (SCANVAC, Vassingerod, Denmark) and then separated and quantified at room temperature on an Agilent HPLC system (Santa Clara, CA, United States) equipped with a C18 reverse-phase column (4.6 mm × 150 mm, particle size of 5 μm) and an UV light detector (230 nm). The mobile phase consisted of HPLC-grade methanol (eluent A) and water (eluent B), and the elution procedure is listed in Table 1 at a flow rate of 1 mL⋅min^–1^. Identification and quantification of putrescine, spermidine and spermine in each sample were achieved by comparing each peak retention time and peak area with the standards, which were conducted in parallel to leaf samples (Supplementary Figure 1). Polyamine contents were calculated using standard curves with commercial standards and a correction for recovery after the extraction procedure.

**TABLE 1:** Elution procedure of polyamines separation in watermelon leaves.

Time(min)	Methanol (eluent A)	Water (eluent B)
0	45%	55%
15	95%	5%
17	100%	0%
18	45%	55%
25	45%	55%

### RNA Extraction and Quantitative Real-Time Polymerase Chain Reaction Analysis

Total RNA was extracted from the fresh leaves by using Tranzol (TransGen Biotech, Beijing, China). The HiScript II One Step RT-PCR Kit (Vazyme Biotech, Beijing, China) was used to perform reverse transcription with 1 μg of RNA according to the manufacturer’s instructions. Gene-specific primers were designed using watermelon coding DNA sequences (v2) obtained from the Cucurbit Genomics Database^1^. Primers were designed using the GenScript bioinformatics tools online^2^. These primers were used for amplification (Supplementary Table 1). qRT-PCR was performed on an ABI 75000 Real-Time polymerase chain reaction (PCR) machine (Applied Biosystems, Foster City, CA, United States) with the following amplification program: 40 cycles of 94°C for 15 s, 60°C for 15 s, and 72°C for 15 s. The melting curve was recorded after 40 cycles to verify the primer specificity. The *ClACT* gene was used as an internal control of watermelon plants (Kong et al., 2014). The relative quantization of gene expression was calculated using the 2^–ΔΔCT^ method (Livak and Schmittgen, 2001).

### RNA-seq Library Preparation and Illumina Sequencing

Total RNA was isolated using TRIzol^®^ reagent (Plant RNA Purification Reagent for plant tissue) according the manufacturer’s instructions (Invitrogen) and genomic DNA was removed using DNase I (TaKara). Then, RNA quality was determined by 2100 Bioanalyser (Agilent) and quantified using ND-2000 (NanoDrop Technologies). Only high-quality RNA (OD260/280 = 1.8–2.2, OD260/230 ≥ 2.0, RIN ≥ 6.5, 28S:18S ≥ 1.0, > 1 μg) was used to construct the sequencing library (Supplementary Table 2). RNA-seq transcriptome library was prepared following the TruSeq™ RNA sample preparation kit from Illumina (SanDiego, CA) using 1 μg of total RNA. RNA-seq libraries were sequenced with the Illumina HiSeq™ xten/NovaSeq 6000 sequencer by Majorbio (Shanghai, China), and the genome reference was the watermelon (97103) v2 genome (see text footnote 1). The original data set was deposited into CNGB Sequence Archive (^3^ accession no. CNP0002368). Data were analyzed online^4^.

### Statistical Analysis

The experiment was conducted using a randomized complete block design. The column diagram was made using OriginPro 7.5. The heatmap of correlation analysis was by TBtool (Chen et al., 2020). Statistical analysis of the bioassays was performed using the SAS statistical package. Differences among the treatment means were analyzed using Tukey’s test (*P* < 0.05).

## Results

### Pumpkin Rootstock Grafting Enhances Chilling Tolerance of Watermelon

**FIGURE 1:**
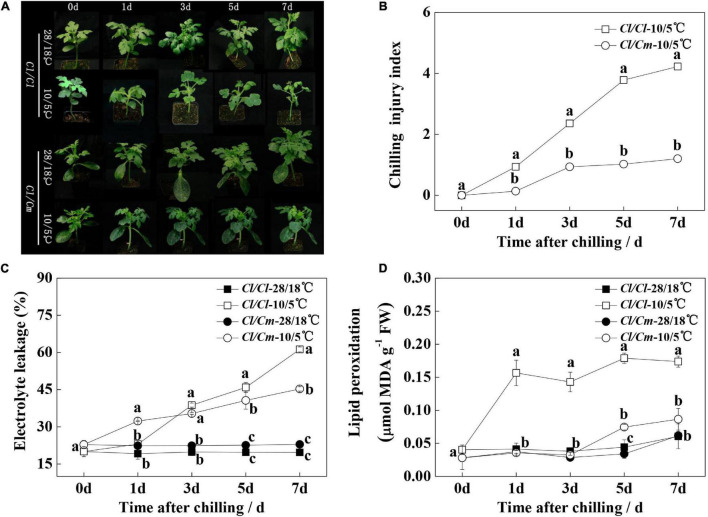
Effects of pumpkin rootstock on the chilling tolerance of watermelon seedlings. **(A)** Representative images of chilling phenotypes. **(B)** Chilling injury index. **(C)** Electrolyte leakage. **(D)** Malondialdehyde (MDA) content. Values are means ± SE (*n* ≥ 4). Different letters indicate the significant differences among various treatments at the same time (*P* < 0.05, one-way ANOVA).

The chilling phenotype and injury symptoms were monitored within 1-week of chilling stress (Figure 1). The seedlings under chilling stress showed wilting and shrinking gradually. The chilling index (CI) continued to increase in *Cl/Cl*, and the *Cl/Cm* seedlings exhibited less severe wilting and shrinking than the *Cl/Cl* seedlings. After 7 days of treatment, the CI values of *Cl/Cl* and *Cl/Cm* were 4.2 and 1.2, respectively (Figure 1B). This phenotypic performance showed that *Cl/Cm* exhibited better chilling-stress tolerance than *Cl/Cl*. In addition, EL and MDA content were measured to investigate cell membrane integrity. The EL of the *Cl/Cl* seedlings increased to 61.3% after 7 days of chilling stress, which was 2.01 times higher than that of the control. The *Cl/Cm* seedlings under chilling stress exhibited less EL. The MDA contents of the *Cl/Cl* and *Cl/Cm* seedlings were significantly higher than those of their control seedlings, but pumpkin grafted alleviated the increase trend of MDA content. These results indicate that *Cl/Cl* and *Cl/Cm* watermelon seedlings respond to chilling stress differently, and rootstock grafting apparently enhances the chilling tolerance of watermelon.

### Effects of Pumpkin Rootstock Grafting on Polyamine Metabolism in Response to Chilling Stress

**FIGURE 2:**
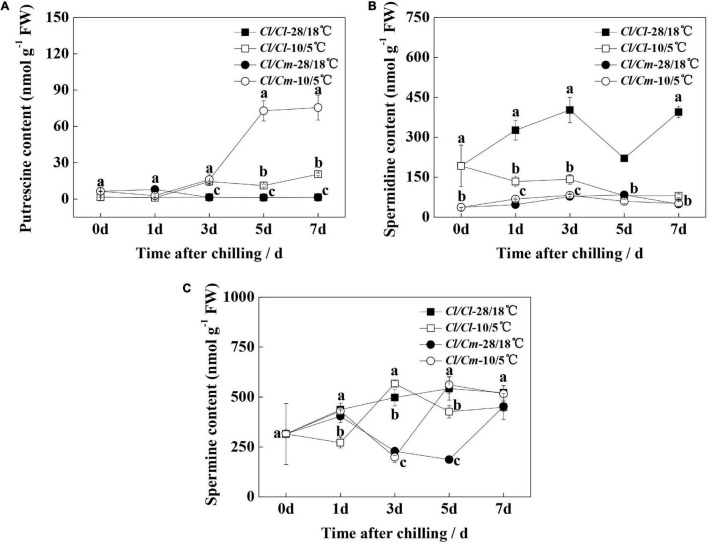
Effects of pumpkin rootstock on polyamine accumulation in watermelon leaves under chilling stress. **(A)** Content of putrescine. **(B)** Content of spermidine. **(C)** Content of spermine. Values are means ± SE (*n* = 4). Different letters indicate the significant differences among various treatments at the same time (*P* < 0.05, one-way ANOVA).

We examined the free polyamine contents during chilling stress to determine the involvement of polyamines in the chilling stress response of watermelon (Figure 2). Results showed that the spermidine content in the leaves of grafted rootstock seedlings decreased, but putrescine and spermidine contents did not change significantly under the 28°C/18°C condition, which might be because grafting inhibited the metabolism of putrescine to spermidine. The putrescine content in the leaves of *Cl/Cl* seedlings slightly increased upon chilling stress, whereas that in the leaves of *Cl/Cm* seedlings significantly increased after 5 days of chilling stress (Figure 2A). Spermidine content showed a constant decline in the *Cl/Cl* seedlings within 7 days of chilling stress, whereas no significant changes in this content were observed in the chilling-stressed or non-stressed *Cl/Cm* seedlings (Figure 2B). Moreover, spermine content showed no definite change characteristics in the *Cl/Cl* and *Cl/Cm* seedlings under chilling stress (Figure 2C). These results suggest that putrescine may be involved in the chilling tolerance of watermelon induced by pumpkin rootstock grafting.

### Inhibition of Putrescine Synthesis Impairs Chilling Tolerance in Pumpkin Rootstock-Grafted Watermelon Seedlings

**FIGURE 3:**
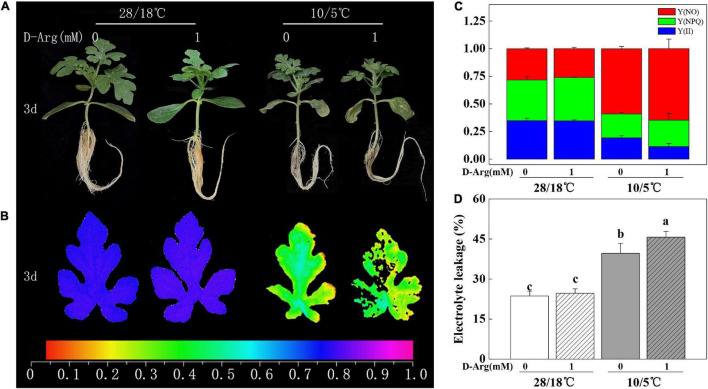
Effects of putrescine synthesis on pumpkin rootstock-induced chilling tolerance in watermelon seedlings. **(A)** Representative images of chilling phenotypes. **(B)** Images of the maximum quantum yield of PS II (*Fv/Fm*). **(C)** Fraction of Y(II), Y(NPQ) and Y(NO). **(D)** Electrolyte leakage. The color gradient of the images in *Fv/Fm* provided at the bottom of this figure B ranges from 0 (black) to 1.0 (purple). Values are means ± SE (*n* ≥ 4) and different letters on the histograms indicate significant differences (*P* < 0.05, one-way ANOVA). *Cl/Cm* seedlings were spray pretreated with 1 mM D-arginine or water (Wu et al., 2016), 12 h before exposure to chilling treatment for 3 days.

We investigated whether or not putrescine is implicated in the enhanced chilling tolerance of *Cl/Cm* seedlings. For this purpose, the *Cl/Cm* seedlings were pretreated with 1 mM D-arginine or water prior to chilling exposure. Results showed that the D-arginine-treated *Cl/Cm* seedlings did not change significantly under the 28°C/18°C condition. After the chilling treatment, the phenotype of the D-arginine-treated *Cl/Cm* seedlings showed severer damages than the control seedlings (Figure 3). The *Fv/Fm* of the D-arginine treated *Cl/Cm* seedlings significantly reduced compared with that of the control under chilling stress, although no obvious difference was observed under normal conditions. PS II condition and EL confirm the role of putrescine in pumpkin rootstock-induced chilling tolerance. These results suggest that plants pretreated with 1 mM D-arginine suffer more serious damage after chilling stress, implying that putrescine contributes to the enhanced chilling tolerance of *Cl/Cm* seedlings.

**FIGURE 4:**
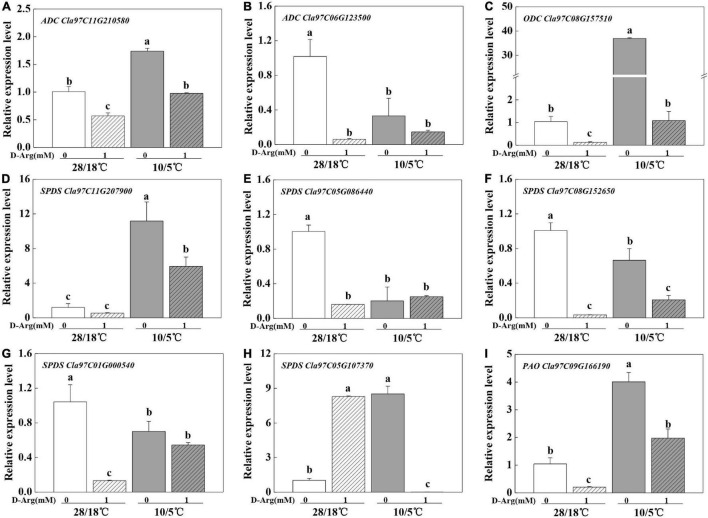
Effects of inhibition of putrescine synthesis on the expression of key genes in polyamine synthesis and metabolism in *Cl/Cm* plants. **(A)** ADC, Cla97C11G210580. **(B)** *ADC, Cla97C06G123500*. **(C)** *ODC, Cla97C08G157510*. **(D)** *SPDS, Cla97C11G207900*. **(E)** *SPDS, Cla97C05G086440*. **(F)** *SPDS, Cla97C08G152650*. **(G)** *SPDS, Cla97C01G000540*. **(H)** *SPDS, Cla97C05G107370*. **(I)** *PAO, Cla97C09G166190*. Values are means ± SE (*n* = 3) and different letters on the histograms indicate significant differences (*P* < 0.05, one-way ANOVA).

We measured the expression of the genes involved in polyamine synthesis and metabolism upon chilling treatment. The results indicated that the expression levels of *arginine decarboxylase (ADC, Cla97C11G210580), ornithine decarboxylase (ODC, Cla97C08G157510* and *Cla97C11G207900), spermidine synthase (SPDS)*, and *PAO (Cla97C09G166190)* were up-regulated (Figure 4). Meanwhile, 1 mM D-arginine treatment inhibited the expression of *ADC* and *ODC*. In addition, putrescine content increased in parallel with *ADC* expression (Figures 2A, 4A). However, the expression of other genes and the contents of other polyamines showed either irregular patterns or no change. Taken together, the results suggest that the up-regulation of *ADC* expression and the increase in putrescine content participate in pumpkin-induced chilling tolerance.

### Transcriptome Analysis Implies an Important Role of the *Arginine Decarboxylase Gene* in the Regulation of Pumpkin Rootstock-Induced Chilling Tolerance in Watermelon

**FIGURE 5:**
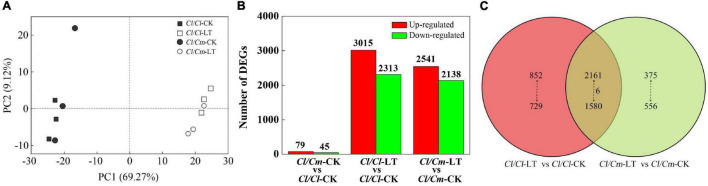
Global transcriptome responses of grafted plants to chilling stress. **(A)** Principal component analysis (PCA) showing the divergence of the respective transcriptomes in response to chilling stress. **(B)** Number of DEGs under different treatments. **(C)** Venn diagrams showing the overlap among DEGs in each treatment.

A summary of RNA-Seq data is shown in Additional file: Supplementary Table 3. Results showed that the sequencing depth and quality were sufficient and reliable for the transcriptome coverage in watermelon. A high correlation was found between biological replicates (*R*^2^ > 0.99) for all treatments (Supplementary Figure 2), which indicated that the biological replicates showed high similarity and reliability in this study. Principal component analysis (PCA) of the transcriptomic data showed that the gene expression profiles of the three independent biological replicates clustered together while separated with different treatments, which indicated that the gene expression differences were significant between the cold-stressed and non-stressed plants (Figure 5A). The PCA indicated 78.39% of total variance for the data set (69.27% for PC1 and 9.12% for PC2). The first component (PC1) separates the control temperature and the chilling treatment, whereas the second component (PC2) separates the two grafted rootstock varieties. The results showed that were mainly separated by PC1, reaching 69.27% variance contribution. This result suggests that the gene expression of the watermelon leaves profoundly changed after the chilling stress.

The transcriptome profiles were analyzed to identify the response of DEGs to chilling stress and pumpkin grafted. At control temperature, 124 (79 up- and 45 down- regulated) genes were differentially expressed in the *Cl/Cm* plants as compared with the *Cl/Cl* plants. After chilling treatment, a clear separation was observed from the control plants. Interestingly, *Cl/Cl* and *Cl/Cm* showed similar changes under chilling stress but became progressively divergent for gene expression alterations in *Cl/Cl* and *Cl/Cm* after 12 h of chilling stress when compared with their respective controls. A total of 5328 (3015 up- and 2313 down- regulated) and 4679 (2541 up- and 2138 down- regulated) chilling-responsive genes were identified in *Cl/Cl* and *Cl/Cm*, respectively (Figure 5B). Venn diagram showed that 1581 chilling-responsive genes (852 up- and 729 down- regulated) were exclusively identified in the *Cl/Cl* seedlings, whereas 931 chilling-responsive genes (375 up- and 556 down- regulated) were uniquely observed in *Cl/Cm*. In addition, 3747 genes (2161 up- and 1580 down- regulated, and 6 of them had opposite expression trends) were commonly regulated by chilling stress in the *Cl/Cl* and *Cl/Cm* seedlings (Figure 5C). To verify the RNA-seq-based gene expression levels, 16 DEGs with differential expression patterns were randomly selected from the *Cl/Cl* and *Cl/Cm* DEGs for qRT-PCR analysis. As expected, the expression of these genes verified by qRT-PCR showed the same expression patterns as those in DEG analysis (Supplementary Figure 3). These results indicate that the pumpkin-grafted seedlings show different responses to chilling stress.

**FIGURE 6:**
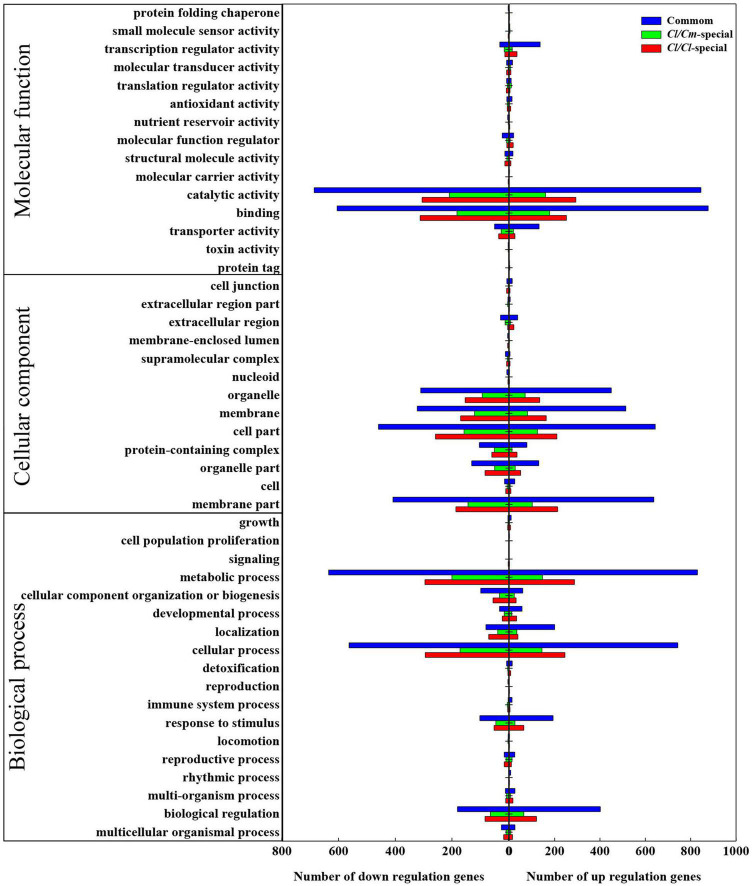
Gene ontology functional analysis of DEGs in *Cl/Cl*-LT and *Cl/Cm*-LT libraries. The x-axis indicates the percentage of up- or down- regulated genes in *Cl/Cl* or *Cl/Cm* libraries under chilling stress in each GO term. The y-axis shows the names of functional categories.

The gene ontology (GO) term enrichment of the common and specific DEGs was analyzed to determine the similarities and differences in chilling-induced transcriptomes between *Cl/Cl* and *Cl/Cm* (Figure 6). A total of 6253 DEGs (3741 commonly regulated, 1581 in *Cl/Cl* and 931 in *Cl/Cm* uniquely regulated) between the control and chilling stress-treated *Cl/Cl* and *Cl/Cm* watermelon seedlings were categorized into 46 functional groups using GO classifications (Figure 6 and Supplementary Table 4). For the molecular function category, catalytic activity process and binding process response to stimulus were the main groups. For the cellular components category, cell part, membrane part, organelle part, and membrane were the main groups. In the biological process category, metabolic process, cellular process, and biological regulation were the main groups. For similarities, GO results were observed between *Cl/Cm* and *Cl/Cl* in either up-regulated or down-regulated DEGs, and *Cl/Cm* significantly reduced the number of DEGs in each biological pathway. These results indicate that the majority of DEGs responding to chilling stress are involved in the catalytic activity process, cell part and metabolic process. Thus, chilling stress treatment mainly affects physiological metabolism in grafted watermelon seedlings.

**FIGURE 7:**
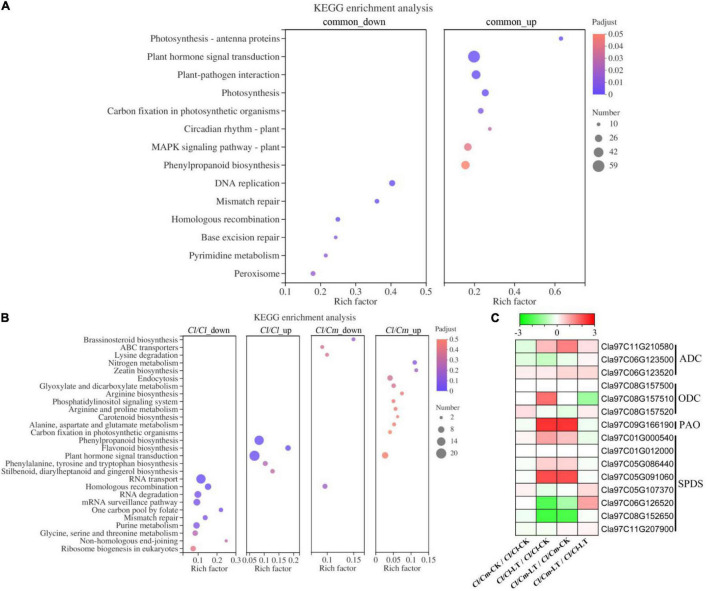
Kyoto encyclopedia of genes and genomes (KEGG) enrichment analysis of DEGs and expression of plant synthesis polyamine-related genes after in *Cl/Cl* and *Cl/Cm* plants. **(A)** Significantly enriched KEGG pathways of common up- and down- regulated genes in response to chilling stress. **(B)** Significantly enriched KEGG pathways of specific up- and down- regulated genes in self- and pumpkin-grafted watermelon in response to chilling stress. **(C)** Effects of chilling tolerance pumpkin grafting on synthesis polyamine genes. Dot color and size indicate the Q-value and gene number, respectively, as shown on the right of panels **(A,B)**. Colors of the boxes represent up-regulated (red) and down-regulated (green) genes. The value is the log_2_ fold-change [log_2_(FC)] of each gene (Panel **C)**.

Biological pathway enrichment analysis was performed by identifying the metabolic pathways or signal transduction pathways that were significantly enriched in the DEGs to gain insights into the biological functions of the DEGs (Figures 7A,B). The genes with common differential expression were mainly enriched in photosynthesis-antenna proteins, plant hormone signal transduction and plant-pathogen interaction, and most of the genes with significant enrichment were up-regulated. Plant hormone signal transduction and homologous recombination were the most common significantly enriched terms for uniquely regulated DEGs in *Cl/Cl* and *Cl/Cm* (Figure 7B). Kyoto encyclopedia of genes and genomes (KEGG) analysis showed that the up-regulated DEGs in the *Cl/Cl* plants were highly enriched in plant hormone signal transduction, phenylpropanoid biosynthesis, and flavonoid biosynthesis. Meanwhile, the main significantly enriched terms of the up-regulated DEGs in *Cl/Cm* were different from those in *Cl/Cl*. Arginine synthesis and arginine and proline metabolism were significantly enriched in pumpkin rootstock uniquely regulated DEGs. Combined with physiological results, the putrescine pathway may be involved in pumpkin rootstock grafting-induced chilling tolerance.

Further analysis of polyamine metabolism pathway genes showed that the *ADC*, *ODC*, *SPDS*, *PAO* genes were up-regulated in response to chilling stress (Figure 7C). Moreover, *ADC* was further up-regulated. The results indicated that polyamine synthesis-related genes were involved in chilling stress response, and *ADC (Cla97C11G210580)* was the key gene up-regulated to promote putrescine accumulation in pumpkin-grafted seedlings.

## Discussion

Chilling is a major environmental stress that has reduced agricultural production in the last few decades (Zhang J. et al., 2016). Watermelon seedling is sensitive to chilling stress for originated from Sudanese in the tropics (Renner et al., 2021). A short period of chilling stress can increase the CI value (Xu et al., 2016; Shi et al., 2019). Despite the widespread use of grafting in horticultural crops, the mechanism by which scion-rootstock interaction regulates plant tolerance to chilling stress needs further investigation. In the present study, the putrescine biosynthesis-associated gene *ADC* was significantly up-regulated in the *Cl/Cm* plants relative to the *Cl/Cl* plants under chilling stress. The data presented here also provide evidence that the pumpkin rootstock increases putrescine biosynthesis in scion leaves when exposed to chilling stress. Inhibiting putrescine accumulation in *Cl/Cm* plants under chilling stress decreases chilling tolerance of *Cl/Cm*. This result was confirmed by transcriptome data.

### Transcriptional Regulation of Pumpkin Rootstock-Induced Watermelon Responses to Chilling Stress

The response to chilling stress in plants is a process by which plants adapt to the environment through gene transcriptional regulation. Previous studies showed that grafting onto pumpkin rootstock changes gene expression and protein accumulation mode, which are involved in multiple biological functions (Xu et al., 2016; Zhang Z. et al., 2016; Shi et al., 2019). The results of the present study suggest that pumpkin rootstock grafting attenuates the transcriptional response of watermelon to chilling stress (Figure 5B). A limited number of DEGs were detected in the pumpkin-grafted watermelon seedlings under normal growth conditions, suggesting that rootstock caused limited change in transcription levels. However, the number of DEGs increased dramatically in the presence of chilling stress, indicating the importance of transcriptional reprogramming in the enhanced chilling tolerance (Figure 5C). Concurrently, the number of up-regulated genes was significantly greater than the number of down-regulated genes, potentially because chilling stress activates the expression levels of genes to promote biological processes to improve chilling tolerance. Strikingly, the number of DEGs was smaller in the pumpkin-grafted plants, which agree with previous studies (Xin et al., 2013; Xu et al., 2016). Thus, pumpkin rootstock grafting reduced the sensitivity of watermelon seedlings to chilling stress and modified gene expression patterns at the transcriptome level response to chilling stress.

### Putrescine Synthesis Participates in Chilling Tolerance Induced by Pumpkin Rootstock Grafting

Plants respond to low temperature by producing various secondary metabolites, among which polyamines play important roles in adapting to environment (Tsaniklidis et al., 2020). Polyamines and metabolism-related genes and enzymes in plants respond to abiotic stress through polyamine accumulation, which is an effective strategy to protect plants against abiotic stress (Shi and Chan, 2014; Liu et al., 2015; Kou et al., 2018; Shahid et al., 2018). Grafting can enhance biotic and abiotic tolerance by increasing the content of polyamines, which strengthens the antioxidative defense system and regulating the ROS homeostasis and stabilization of membranes (Zhang Z. et al., 2010; Duan et al., 2017; Shahid et al., 2018). Previous studies have found that polyamines, especially putrescine (Xu et al., 2011; Song et al., 2014b; Li M. et al., 2021), function to mitigate cold-induced oxidative damages in plants (Lee, 1997; Song et al., 2014a; Gu et al., 2019; Gao et al., 2020; Tsaniklidis et al., 2020). Putrescine biosynthesis and accumulation mitigate cold-induced oxidative stress by regulating antioxidant systems in plants (Song et al., 2014b; Ding et al., 2021). At present, few studies focused on the regulatory relationship between grafting and *ADC* genes. In the current study, our data demonstrated that pumpkin rootstock grafting played a positive role in putrescine synthesis and accumulation under chilling stress (Figure 2A), consistent with the up-regulated expression of the *ADC* gene (Figures 4, 7). Similarly, an increase in expression of the *ADC* gene and accumulation of putrescine under chilling stress have been reported in *Brassica rapa* (Yin et al., 2020). Moreover, a foliar spray D-arginine experiment further confirmed that putrescine under chilling stress in *Cl/Cm* inhibits ADC activity to modulate the metabolism of plants and the production of metabolites involved in stress tolerance (Song et al., 2014a,^2021^; Wu et al., 2016; Wang et al., 2019).

Polyamine is a crucial factor in plant response to low temperature (Song et al., 2021). Changes in transcriptome-level changes in *Cl/Cl* and *Cl/Cm* leaves under chilling stress were determined to elucidate the mechanism behind pumpkin-induced chilling tolerance. As expected, uniquely regulated genes in the *Cl/Cm* plants, which were enriched in the putrescine-associated pathways (arginine synthesis, arginine and proline metabolism) (Figure 7), were consistent with the proteome study of grafted watermelon under chilling stress (Shi et al., 2019). The relative mRNA expression levels of *ADC*, *ODC*, and *SPDS* involved in putrescine biosynthesis were significantly up-regulated; the expression of *ADC* in *Cl/Cm* leaves was further up-regulated under chilling stress, which was the main reason for the increased putrescine accumulation in the leaves of *Cl/Cm* plants (Figure 7C). Putrescine signaling is involved in the pumpkin rootstock grafting-induced chilling tolerance and agrees with previous results (Shen et al., 2000; Liu et al., 2015; Kou et al., 2018; Li M. et al., 2021). In addition, the content of polyamines in plants can be used as an important index to measure their resistance to stress because polyamine-rich plants usually show high resistance to stress (Shi and Chan, 2014; Kou et al., 2018; Song et al., 2021). However, no noteworthy regularly were found in the spermine and spermidine contents. Similar results indicated that putrescine’s downstream product spermidine does not increase with putrescine content (Kou et al., 2018). Thus, the present study suggests that the specific increase in putrescine content may be closely related to the stress signal in response to chilling stress and may slow the damage and enhance the chilling tolerance in pumpkin-grafted seedlings.

Polyamines are polycations that covalently bind negatively charged macromolecules, such as DNA, RNA, and proteins, as structural supports; regulate different cellular processes; and stabilize the plasma membrane of the cells to survive stress (Kasukabe et al., 2004; Igarashi, 2006; Chen et al., 2019). Increasing evidence supported that the accumulation of polyamines is related to high chilling resistance in various fruits and vegetables (Nair and Singh, 2004; Zhang X. et al., 2012). This study found that self-grafted seedlings with low putrescine contents develop CI symptoms. Meanwhile, grafting of pumpkin rootstock promotes the accumulation of putrescine, which might help maintain the normal function of membrane and reduce the CI value under chilling stress and enhance chilling tolerance (Figures 1, 2). This result is consistent with the result that putrescine participates in the regulation of chilling tolerance (Jiao et al., 2021). The reason for its enhancement of chilling tolerance might be that putrescine stabilizes cell components and might act as a signal that triggers the anti-oxidant system to reduce MDA accumulation and comprehensively enhance chilling tolerance (Igarashi, 2006; Ding et al., 2021). Thus, ADC-catalyzed putrescine synthesis may promote the chilling tolerance in pumpkin-grafted seedlings, and these results provide useful information for further research on the molecular mechanism by which ADC participates in chilling tolerance in grafting-induced chilling tolerance.

### Pumpkin Rootstock Grafting Is an Effective Method to Increase the Chilling Tolerance of Watermelon

Grafting onto tolerance rootstock can enhance biotic and abiotic tolerance (Schwarz et al., 2010; Zhang Z. et al., 2010; Duan et al., 2017; Nawaz et al., 2017; Shahid et al., 2018; Shireen et al., 2020). Pumpkin is a widely used watermelon rootstock in enhancing crop stress resistance and improving crop quality (Xu et al., 2016; Nawaz et al., 2017, 2018; Shi et al., 2019). In the present study, we found that using pumpkin as rootstock can enhance the tolerance of watermelon scion under chilling stress, as evidenced by low CI value, EL and MDA content accumulation in *Cl/Cm* plants compared with the *Cl/Cl* plants under chilling stress (Figure 1). This result is consistent with those of previous studies (Liu et al., 2003; Xu et al., 2016; Shi et al., 2019; Li H. et al., 2021). Thus, pumpkin rootstock can increase the chilling tolerance of grafted plants (Xu et al., 2016; Shi et al., 2019; Li H. et al., 2021).

## Conclusion

**FIGURE 8:**
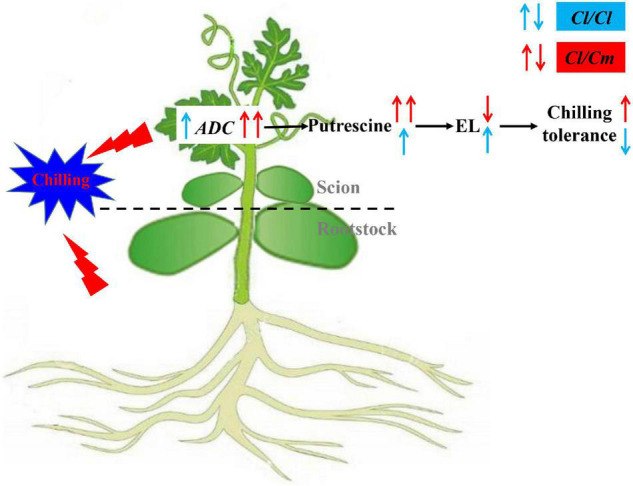
Proposed model for putrescine involved in pumpkin grafted induced chilling tolerance in watermelon plants. Pumpkin rootstock grafting increased *ADC* expression and putrescine accumulation, decreased electrolyte leakage under chilling stress, thus increasing chilling tolerance. Red arrows represent pumpkin rootstock-increased chilling tolerance; Blue arrows represent watermelon rootstock-caused chilling sensitivity.

Transcriptomic and physiological analyses provided new insights into chilling tolerance in pumpkin-grafted watermelon seedlings, and a working model of the pumpkin-grafted regulatory network involved in watermelon seedlings was proposed (Figure 8). This model supports the concept that the pumpkin rootstock grafting-induced chilling tolerance of watermelon through transcription-associated alterations, especially up-regulating the expression level of *ADC* and promoting the synthesis of putrescine under chilling stress, mitigates cold-induced oxidative stress, and ultimately improves the chilling tolerance of pumpkin-grafted watermelon plants. This study provides primary analysis about the molecular mechanisms underlying grafted-mediated chilling tolerance. However, the factor responsible for the up-regulation of *ADC* in pumpkin-grafted seedlings to regulate polyamine level remains to be determined. Therefore, further studies should be conducted to provide genetic evidence of the involvement of putrescine in chilling tolerance. Other mechanisms underlying grafting-mediated chilling tolerance should also be explored to understand the relationship between grafting and chilling signaling.

## Data Availability Statement

The original contributions presented in the study are publicly available. This data can be found here: https://db.cngb.org/search/project/CNP0002368/.

## Author Contributions

JL, FC, YH, and ZB conceived and designed the experiments and revised the manuscript. JL performed the experiments, analyzed the data, and wrote the manuscript. All of the authors read and approved the final version of the manuscript.

## Conflict of Interest

The authors declare that the research was conducted in the absence of any commercial or financial relationships that could be construed as a potential conflict of interest.

## Publisher’s Note

All claims expressed in this article are solely those of the authors and do not necessarily represent those of their affiliated organizations, or those of the publisher, the editors and the reviewers. Any product that may be evaluated in this article, or claim that may be made by its manufacturer, is not guaranteed or endorsed by the publisher.

## Footnotes

1
http://cucurbitgenomics.org/organism/21

2
https://www.genscript.com/tools.html

3
https://www.cngb.org/

4
http://vip.majorbio.com/
