# Supplementary material for: Grafting Watermelon Onto Pumpkin Increases Chilling Tolerance by Up Regulating Arginine Decarboxylase to Increase Putrescine Biosynthesis
Source: Front Plant Sci. 2022 Feb 15;12:812396. doi: 10.3389/fpls.2021.812396 (PMC8886213; doi:10.3389/fpls.2021.812396)
Supplement: Supplementary file 2 [file Data_Sheet_1.pdf]

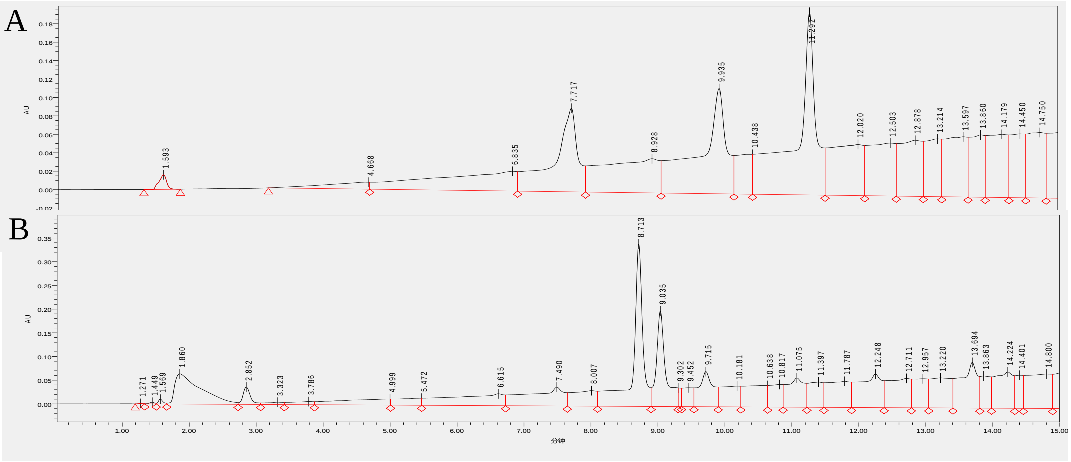

Fig. S1 The diagram of polyamine elution peak in watermelon leaves

A, Standard substance. B, Sample.

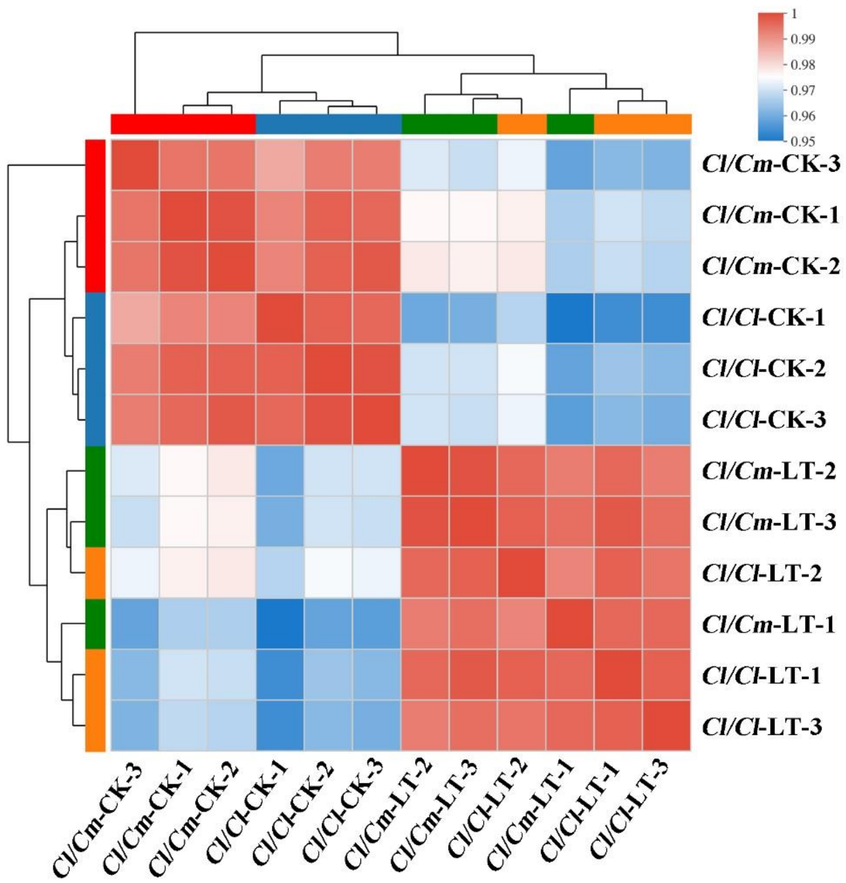

Fig. S2 Hierarchical clustering of 12 samples based on the correlation coefficient (R^2) between each sample.

The colors of the boxes represents the R^2 values.

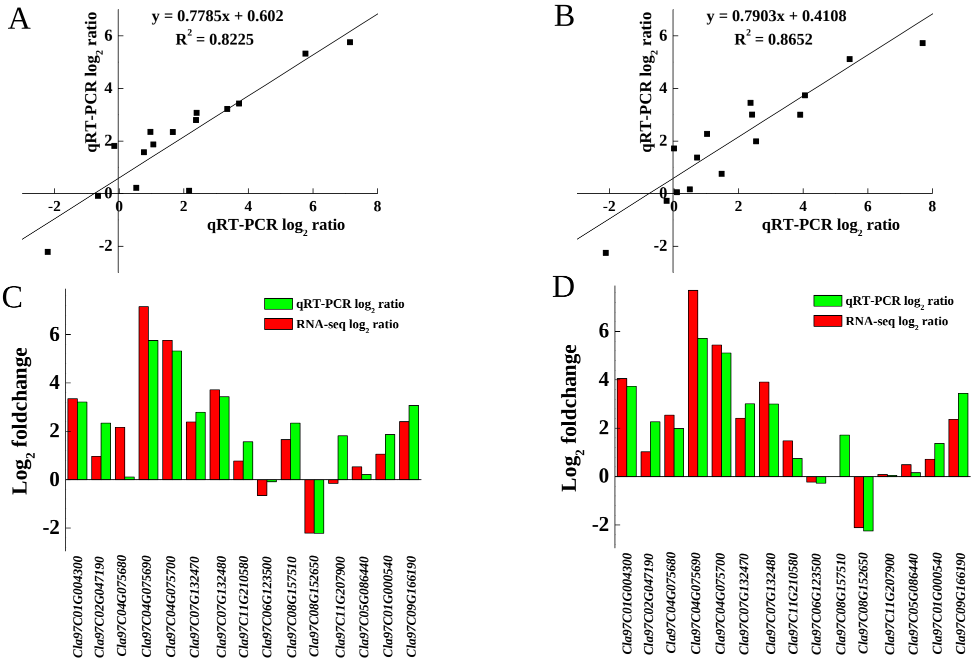

Fig. S3 qRT-PCR validation of transcriptome data

A, Correlation analysis between transcriptome data and qRT-PCR results in self-grafted watermelon seedling. B, Correlation analysis between transcriptome data and qRT-PCR results in rootstock-grafted watermelon seedling. C, The expression level of selected gene analyzed by transcriptome and qRT-PCR in self-grafted watermelon seedling, $\log_2$ ratio=*Cl/Cl*-LT vs *Cl/Cl*-CK. D, The expression level of selected gene analyzed by transcriptome and qRT-PCR in rootstock-grafted watermelon seedling, $\log_2$ ratio=*Cl/Cm*-LT vs *Cl/Cm*-CK.

Tab. S1 The primers for qRT-PCR

**Primer**	**Forward primer (5’-3’)**	**Reverse Primer (5’-3’)**
**ClACTIN*	CCATGTATGTTGCCATCCAG	GGATAGCATGGGGTAGAGCA
PIP-Cla97C01G004300	AATCACCTTCGGTCTGGTGT	CATGGACCCACCGCTAAATG
PIP-Cla97C02G047190	TCATCGCCACTCTCCTCTTC	GCGGTGCAGTAAACAAGGAT
PIP-Cla97C04G075680	TGTCTTGGTGCCATTTGTGG	TAATCTCAGCGCCTAAGCCA
PIP-Cla97C04G075690	TGTACATGGTGGCTCAGTGT	CCGCCGTACTTGTTGTAGTG
PIP-Cla97C04G075700	CCCAATTGGATTCGCAGTGT	TCATCCCAGGGCTTGTCTTT
PIP-Cla97C07G132470	TTCACCGAGAGACAGCCAAT	GGTGGCTCCTTGTAGTCCTT
PIP-Cla97C07G132480	CAACCCAAGCCCTACGAAAG	ATCGGAACATGCGAGTCTCT
ADC-Cla97C11G210580	TTCGGGTGATGCAGAGTGAT	TGCTTGAGGGTCTCGAACAT
ODC-Cla97C08G157510	TACAAATCGGCGGTGTTTGG	GGAGGAGGTGGTAAACCCAT
PAO-Cla97C09G166190	AGGTGTTGGTGGACAATGGA	CAAACCGGATTCGGCTCTTT
Spm-Cla97C01G000540	CGTCCACCTTCACATTGGTG	GCCCAACAGGATCTGAGGAA
ADC-Cla97C06G123500	GGCCGCCATCATTAACTCAG	GCTTGCTGCTGTGCTTCTAA
SPDS-Cla97C05G086440	TGCGACATCGATGAGGAAGT	AATTGGGTCTGCAAGATCGC
SPDS-Cla97C05G107370	GAGGAGATGGCGGTGTCTTA	ATGAAGAGTGACGCGAGGAT
SPDS-Cla97C08G152650	ATGGGAAGATGCAGAGTGCT	TCCACCACGTCCTGATCAAT
SPDS-Cla97C11G207900	TCAAATGGTCGTTTGCCGTT	CACTTTGCAGCTTCCCATCA

Note：**ClACTIN* use as reference genes for watermelon

Tab. S2 RNA quality information of RNA sequencing

Sample name	Content (ng/μl)	OD260/280	OD260/230	RIN	Result
Cl/Cl-CK-1	1650.7	2.16	2.37	7.3	B
Cl/Cl-CK-2	1403.2	2.19	2.37	7.7	B
Cl/Cl-CK-3	681.6	2.24	2.05	7.9	B
Cl/Cm-CK-1	629.7	2.21	1.89	8	A
Cl/Cm-CK-2	1306.7	2.16	2.43	8.1	A
Cl/Cm-CK-3	1017.3	2.21	1.95	7.3	B
Cl/Cl-LT-1	1890.2	2.16	2.12	8.2	A
Cl/Cl-LT-2	1514.3	2.18	2.36	7.2	B
Cl/Cl-LT-3	1662.4	2.18	2.36	6.9	B
Cl/Cm-LT-1	1156.8	2.21	1.8	9	A
Cl/Cm-LT-2	1322.5	2.16	2.42	8.7	A
Cl/Cm-LT-3	1759	2.14	2.38	8.8	A

Tab. S3 Quality control results of transcriptome sequencing

**Sample name**	**Total reads**	**Total mapped**	**Multiple mapped**	**Uniquely mapped**
Cl/Cl-CK-1	52844954	51086708(96.67%)	1524163(2.88%)	49562545(93.79%)
Cl/Cl-CK-2	44475014	42741248(96.1%)	1264741(2.84%)	41476507(93.26%)
Cl/Cl-CK-3	58072410	56026590(96.48%)	1469158(2.53%)	54557432(93.95%)
Cl/Cm-CK-1	61421404	59267407(96.49%)	2401680(3.91%)	56865727(92.58%)
Cl/Cm-CK-2	49571696	47803934(96.43%)	1621369(3.27%)	46182565(93.16%)
Cl/Cm-CK-3	43988082	42593581(96.83%)	1226683(2.79%)	41366898(94.04%)
Cl/Cl-LT-1	45347766	43569254(96.08%)	1856076(4.09%)	41713178(91.99%)
Cl/Cl-LT-2	50327142	48542846(96.45%)	2676952(5.32%)	45865894(91.14%)
Cl/Cl-LT-3	39738226	37990587(95.6%)	2584087(6.5%)	35406500(89.1%)
Cl/Cm-LT-1	50989652	48584784(95.28%)	3635079(7.13%)	44949705(88.15%)
Cl/Cm-LT-2	53619238	51554143(96.15%)	1722177(3.21%)	49831966(92.94%)
Cl/Cm-LT-3	47641514	45596030(95.71%)	1782882(3.74%)	43813148(91.96%)
